# Supplementary material for: CALIPSO lidar level 3 aerosol profile product: version 3 algorithm design
Source: Atmos Meas Tech. Author manuscript; Available in PMC 2021 Jan 27. (PMC7840064; doi:10.5194/amt-11-4129-2018)
Supplement: Supplement [file NIHMS1538708-supplement-Supplement.pdf]

Supplement of Atmos. Meas. Tech., 11, 4129–4152, 2018
https://doi.org/10.5194/amt-11-4129-2018-supplement
© Author(s) 2018. This work is distributed under
the Creative Commons Attribution 4.0 License.

Atmospheric Measurement Techniques
Open Access

Supplement of

CALIPSO lidar level 3 aerosol profile product: version 3 algorithm design

Jason L. Tackett et al.

Correspondence to: Jason L. Tackett (jason.l.tackett@nasa.gov)

The copyright of individual parts of the supplement might differ from the CC BY 4.0 License.

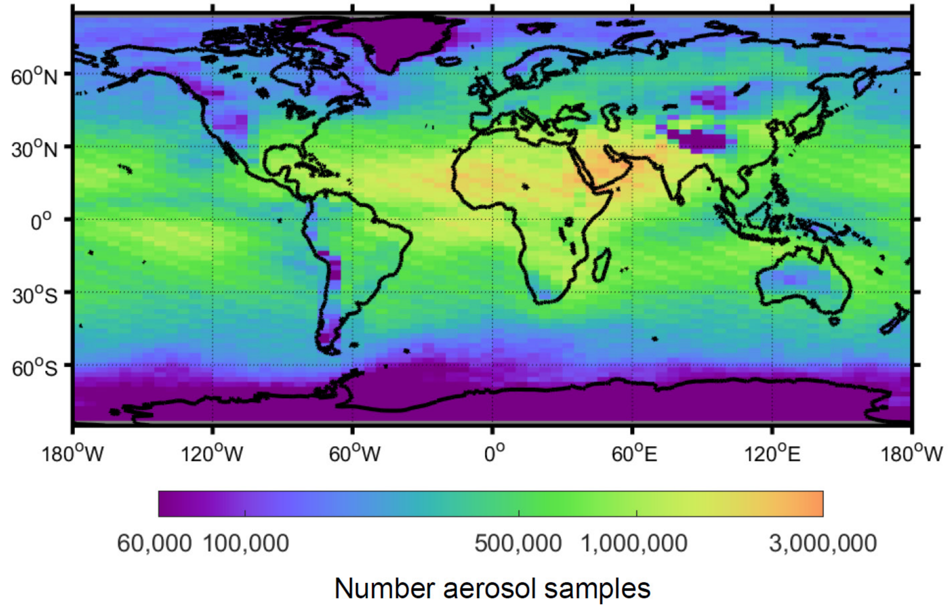

Figure S1. Total number of aerosol samples reported by the level 3 product prior to quality screening for 2007–2016 at day, all-sky.

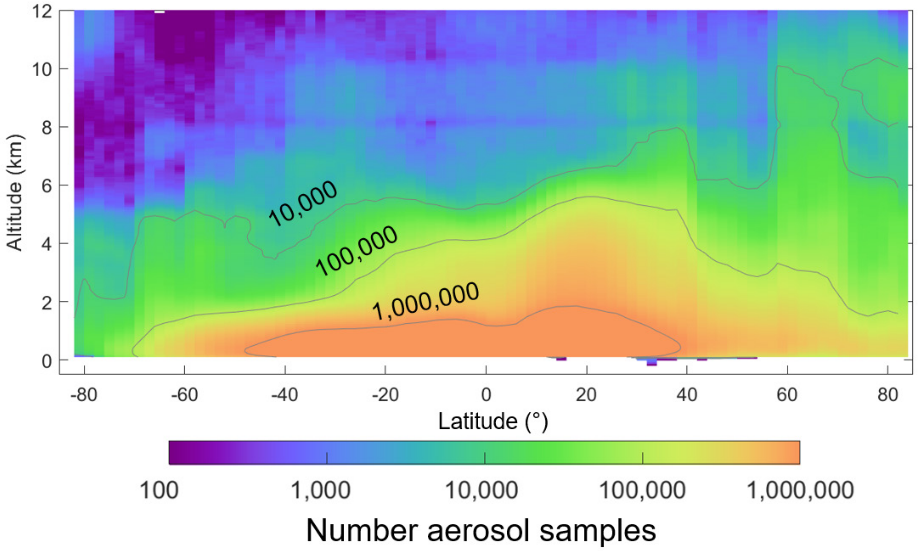

Figure S2. Zonal total number of aerosol samples reported by the level 3 product prior to quality screening for 2007–2016 at day, all-sky.

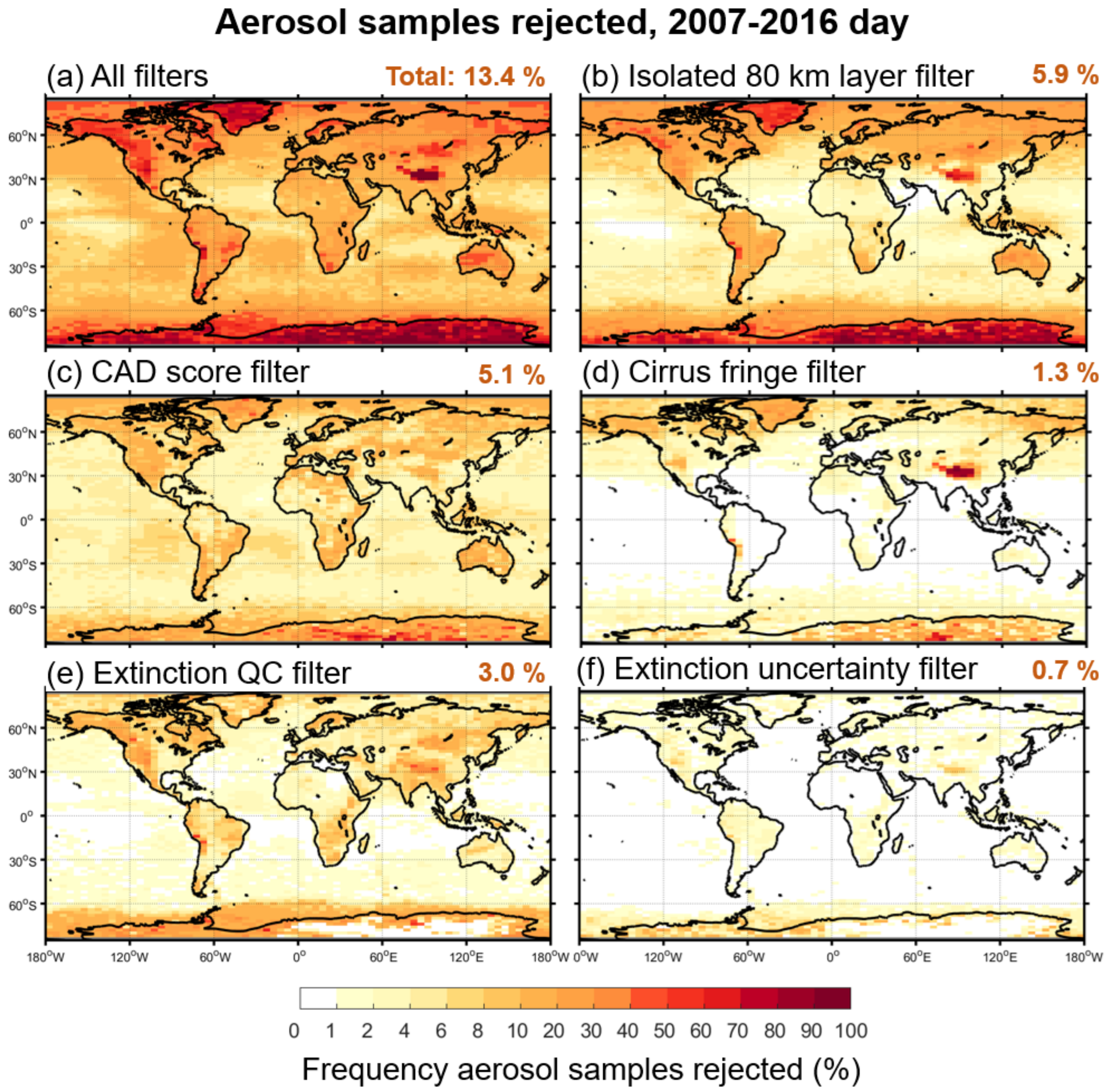

Figure S3. Frequency of aerosol samples rejected by the indicated filter out of all aerosol detected as reported by the level 3 product for 2007–2016 at day, all-sky. Global total rejection frequencies are indicated in the panel titles.

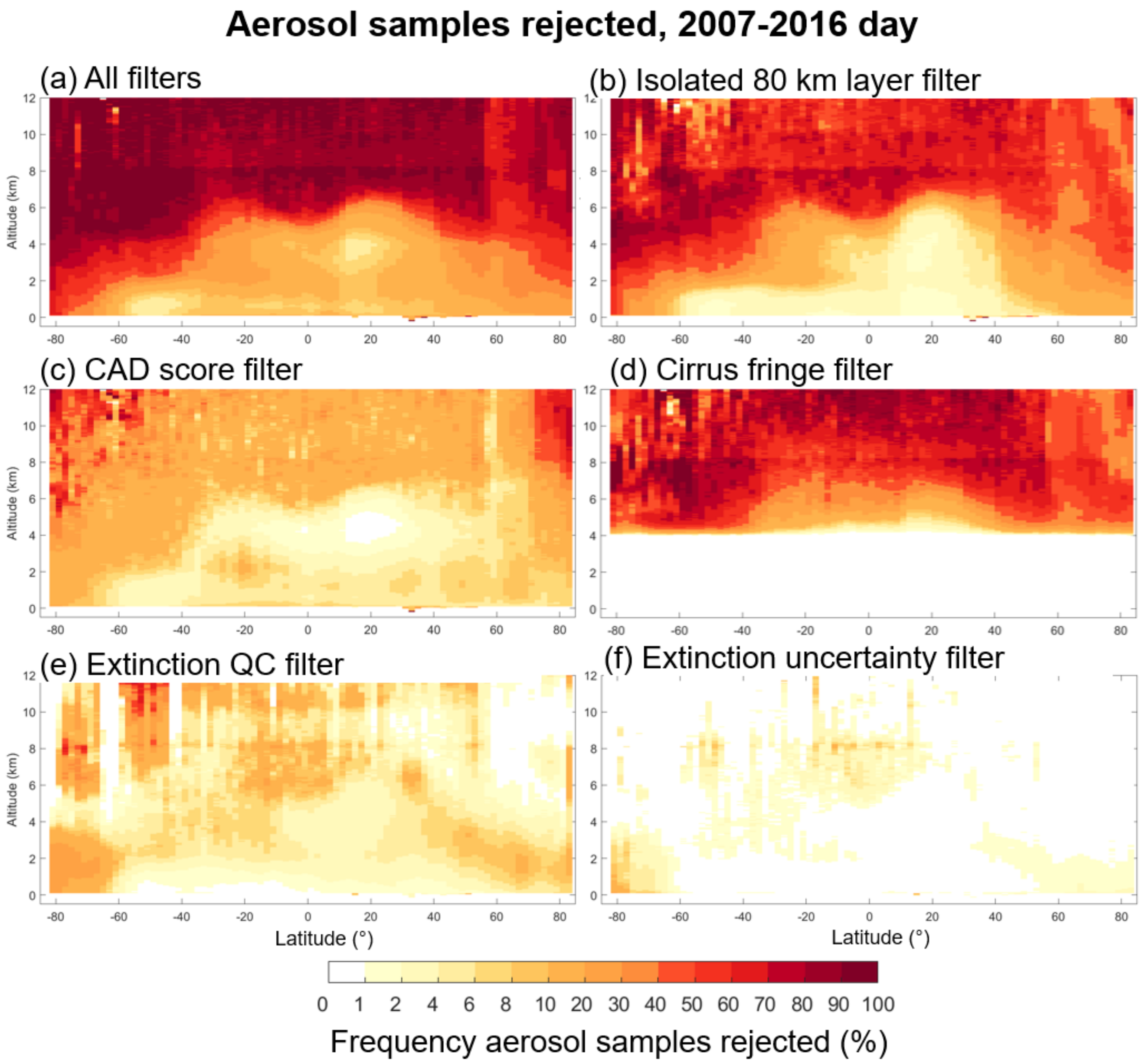

Figure S4. Zonal frequency of aerosol samples rejected by the indicated filter out of all aerosol detected as reported by the level 3 product for 2007–2016 at day, all-sky.

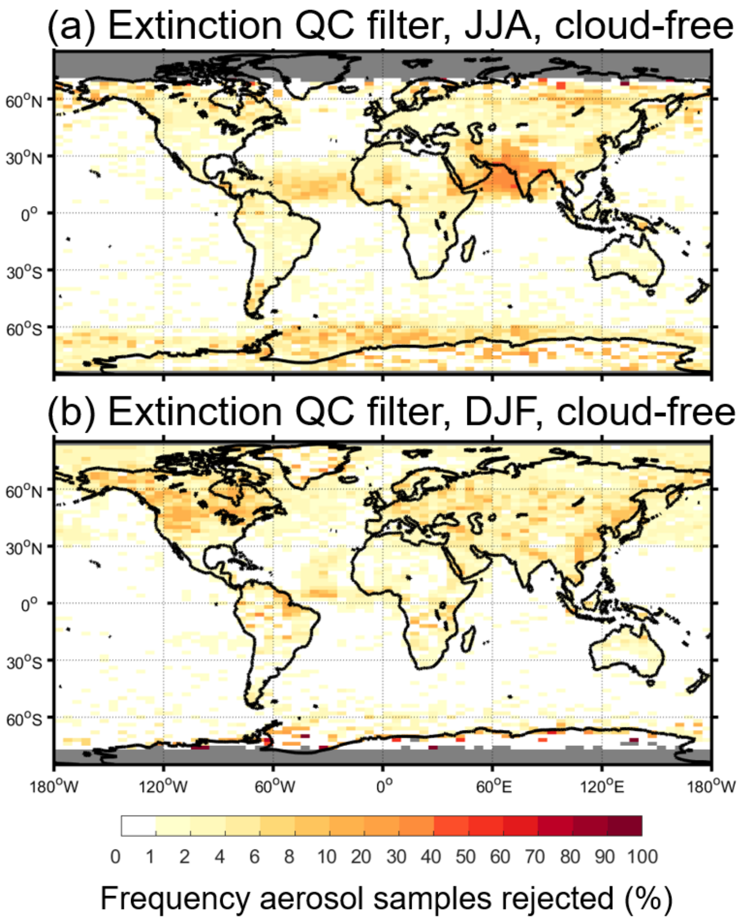

Figure S5. Frequency of aerosol samples rejected by the extinction QC filter out of all aerosol detected for (a) JJA and (b) DJF, 2007–2016 at night, cloud-free.

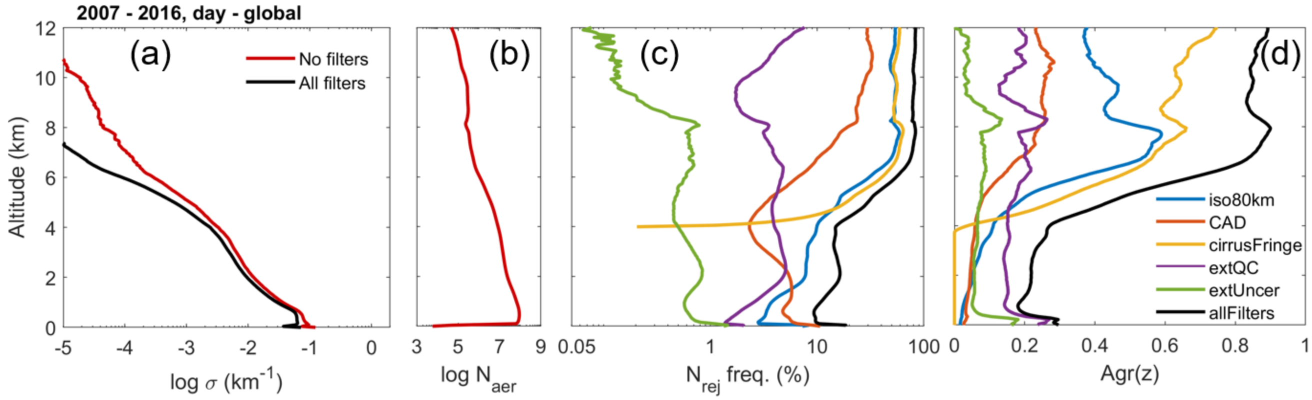

Figure S6. (a) Mean extinction with and without quality filters, (b) number of unfiltered aerosol samples, (c) frequency of aerosol samples rejected, and (d) filter aggressiveness (Eq. (B1)) smoothed vertically over 600 meters for 2007–2016 at day, all-sky.

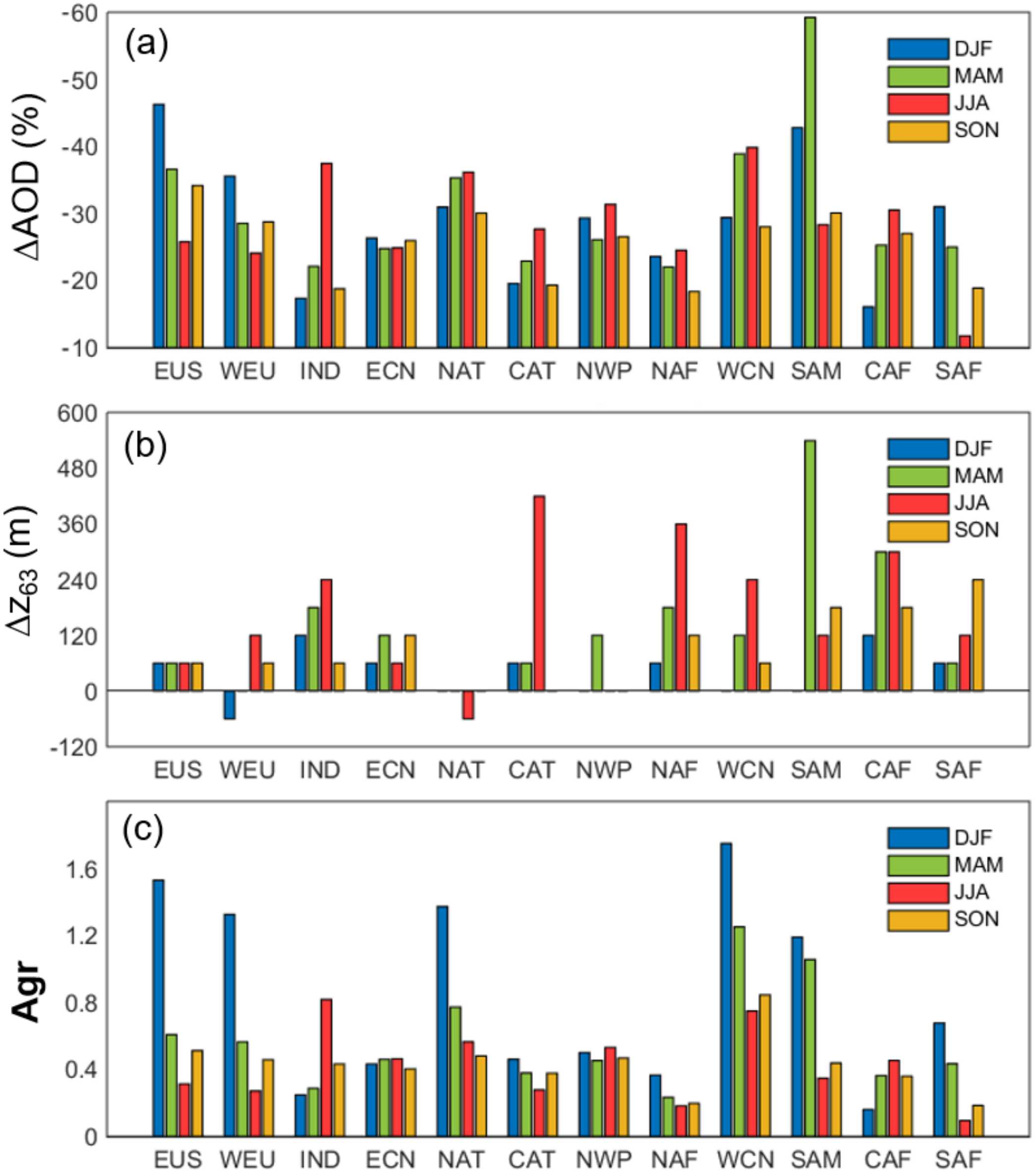

Figure S7. Regional changes in AOD and with no filters compared with all filters: (a) percent reduction in AOD with numbers above the bars indicating mean filtered AOD, (b) difference in 63% extinction scale heights (all filters – no filters; Eq. (B3)), and (c) filter aggressiveness (Eq. (B4)) for 2007–2016 at night, all-sky. Samples at altitudes ≤ 0.039 km are excluded due to low sample counts.

Table S1. Global metrics comparing changes in mean AOD and $\overline{\sigma}$ with no filters against all filters and with each filter applied independently for global ocean and global land for 2007–2016 at night, all-sky: AOD with all filters, ΔAOD = percent change in AOD, Δz_{63} = difference in 63% extinction scale heights (all filters – no filters; Eq. (B3)), *Agr* = aerosol sample-weighted mean of filter extinction impact profile (Eq. (B4)). Samples at altitudes ≤ 0.039 km are excluded due to low sample counts.

	**DJF**				**JJA**			
	AOD	ΔAOD (%)	Δz_{63} (m)	***Agr***	AOD	ΔAOD (%)	Δz_{63} (m)	***Agr***
EUS	0.09	−46	60	1.53	0.15	−26	60	0.31
WEU	0.13	−36	−60	1.32	0.17	−24	120	0.27
IND	0.34	−17	120	0.25	0.46	−37	240	0.82
ECN	0.50	−26	60	0.43	0.42	−25	60	0.46
NAT	0.11	−31	0	1.37	0.06	−36	−60	0.56
CAT	0.17	−20	60	0.46	0.25	−28	420	0.28
NWP	0.14	−29	0	0.50	0.11	−31	0	0.53
NAF	0.21	−24	60	0.37	0.45	−24	360	0.18
WCN	0.55	−29	0	1.75	0.48	−40	240	0.75
SAM	0.15	−43	0	1.19	0.16	−28	120	0.35
CAF	0.42	−16	120	0.16	0.31	−31	300	0.45
SAF	0.17	−31	60	0.68	0.34	−12	120	0.09
	**MAM**				**SON**			
	AOD	ΔAOD (%)	Δz_{63} (m)	***Agr***	AOD	ΔAOD (%)	Δz_{63} (m)	***Agr***
EUS	0.11	−37	60	0.61	0.09	−34	60	0.51
WEU	0.15	−29	0	0.56	0.14	−29	60	0.46
IND	0.42	−22	180	0.29	0.33	−19	60	0.43
ECN	0.45	−25	120	0.46	0.44	−26	120	0.40
NAT	0.08	−35	0	0.77	0.07	−30	0	0.48
CAT	0.21	−23	60	0.38	0.14	−19	0	0.38
NWP	0.15	−26	120	0.45	0.11	−27	0	0.47
NAF	0.34	−22	180	0.23	0.25	−18	120	0.20
WCN	0.43	−39	120	1.25	0.44	−28	60	0.84
SAM	0.09	−59	540	1.06	0.30	−30	180	0.44
CAF	0.35	−25	300	0.36	0.24	−27	180	0.36
SAF	0.15	−25	60	0.43	0.34	−19	240	0.18

	**2007 – 2016**			
	AOD	ΔAOD (%)	Δz_{63} (m)	***Agr***
EUS	0.11	−34	60	0.51
WEU	0.15	−28	60	0.44
IND	0.38	−24	120	0.42
ECN	0.44	−25	120	0.44
NAT	0.08	−33	−60	0.60
CAT	0.19	−23	60	0.35
NWP	0.12	−28	60	0.48
NAF	0.30	−23	180	0.22
WCN	0.46	−34	60	1.01
SAM	0.18	−38	240	0.62
CAF	0.34	−23	180	0.31
SAF	0.27	−18	120	0.23
